# Transduction-mediated horizontal gene transfer in the oral microbiome

**DOI:** 10.3389/fcimb.2015.00012

**Published:** 2015-02-10

**Authors:** Roy H. Stevens

**Affiliations:** Laboratory of Oral Infectious Diseases, Endodontics, Temple UniversityPhiladelphia, PA, USA

**Keywords:** horizontal gene transfer, bacteriophages, oral microbiome, transduction, genetic, oral cavity

The recent article, “The impact of horizontal gene transfer on the adaptive ability of the human oral microbiome” by Roberts and Kreth (2014) provides a valuable review of the mechanisms and consequences of horizontal gene transfer (HGT) in the oral microbiome. It discusses the three well-known mechanisms of HGT (i.e., transformation, conjugation and transduction), as well as the recently described mechanism of vesicle-mediated gene transfer, although the bulk of the attention is given to transformation occurring in oral biofilms. The authors emphasize the potential importance of HGT in the oral microbiome by pointing to the demonstrated transfer of genes (*in vitro*) from transient oral bacteria, unable to colonize oral biofilms, to members of the established oral microbiome community. Thus, the genes (and their consequences) may remain in the oral cavity long after the (bacterial) sources of the genes are gone. In this regard, it should be noted that there have been several recent reports of transient oral infections following the ingestion of certain foods, such as cheeses (Razavi et al., 2007; Kamodyová et al., 2014).

Whilst the magnitude of the bacteriophage load in the oral cavity is acknowledged by the authors, the potential of bacteriophages for HGT via transduction in the oral cavity is, however, not as clearly or accurately described. For example, the authors state that “Studies on saliva have isolated bacteriophages able to lysogenize specific bacterial pathogens such as *Enterococcus faecalis* (Bachrach et al., 2003; Stevens et al., 2009) and *Aggregatibacter actinomycetemcomitans* (Sandmeier et al., 1995; Willi et al., 1997).” While the Bachrach et al paper *did* report on *E. faecalis* phages isolated from saliva, the Stevens et al article reported on bacteriophages induced from *E. faecalis* isolated from infected root canals (NOT saliva), and the source of the *A. actinomycetemcomitans* from which phages were induced in the works by Sandmeier et al, and Willi et al, was subgingival plaque (again, NOT saliva). This is of some significance since it demonstrates that there are multiple niches (not just saliva) harboring bacterial viruses in the oral cavity, and phages in any of these niches could be a vehicle for HGT. Secondly, although HGT may occur by temperate phages via specialized transduction, it is also possible for HGT to occur by virulent phages through generalized transduction. Consequently, “…bacteriophages able to lysogenize…” may not be the only explanation for transduction-mediated HGT. This was not considered in the discussion of bacteriophages and HGT. Further, it is quite curious that the authors state that “…there have been no reported observations of transduction in the oral cavity, or relevant *in vitro* models…” since the title of the Willi et al. article cited in this review is “**Transduction** of antibiotic resistance markers among *Actinobacillus actinomycetemecomitans* strains by temperate bacteriophage Aa phi 23” (emphasis added). This study clearly demonstrated the capacity of bacteriophages (through transduction) to horizontally transfer genes between strains of oral bacteria, at least *in vitro*. Finally, it is customary that the references cited should be the *original* source of the information in question (Annesley, 2011). Reports of *E. faecalis* bacteriophages in the oral cavity (e.g., Natkin, 1967) were published more than 30 years prior to the two articles cited above by the authors. Similarly, isolation of *A. actinomycetemcomitans* bacteriophages was originally reported (Stevens et al., 1982) more than 10 years prior to the articles (Sandmeier et al., 1995; Willi et al., 1997) cited by the authors.

## Conflict of interest statement

The author declares that the research was conducted in the absence of any commercial or financial relationships that could be construed as a potential conflict of interest.
